# Supplementary material for: High hedgehog signaling is transduced by a multikinase-dependent switch controlling the apico-basal distribution of the GPCR smoothened
Source: eLife. 2022 Sep 9;11:e79843. doi: 10.7554/eLife.79843 (PMC9462849; doi:10.7554/eLife.79843)
Supplement: Source code 1. [file elife-79843-code1.zip › 28-04-2022-RA-RC-eLife-79843/Appendix 2 Fiji Macro.docx]

# Appendix 2

**Fiji quantification macro**

nbBand=9;

pourcentApical=0.15;

pourcentBasal=0.10;

run("Set Measurements...", " redirect=None decimal=9");

myImage=getTitle();

selectWindow(myImage);

run("Split Channels");

close();

close();

segmentation("C1-"+myImage);

nbBand = createROI2("C1-"+myImage,"Mask of imgForThreshold",nbBand);

run("Z Project...", "projection=[Average Intensity]");

for(n=0;n<nbBand;n++){

setResult("image",n,myImage);

ApicalBasal("AVG_C1-"+myImage,n,pourcentApical,pourcentBasal);

}

for(n=0;n<nbBand;n++){

roiManager("Select",0);

roiManager("Delete");

}

intensityMap("AVG_C1-"+myImage);

function segmentation(nameImage){

selectWindow(nameImage);

run("Z Project...", "projection=[Average Intensity]");

selectWindow("AVG_"+nameImage);

run("Duplicate...", "duplicate channels=1");

rename("imgForThreshold");

run("Median...", "radius=20 stack");

run("8-bit");

run("Auto Local Threshold", "method=Sauvola radius=128 parameter_1=0.25 parameter_2=256 white");

run("Analyze Particles...", "size=300000-Infinity pixel show=Masks");

run("Invert LUT");

run("Fill Holes");

run("Options...", "iterations=100 count=2 black pad do=Close");

selectWindow("imgForThreshold");close();

}

function createROI2(nameImage,nameMask,nbBand){

selectWindow(nameMask);

run("Create Selection");

selectWindow(nameImage);

run("Restore Selection");

roiManager("Add");

roiManager("Select", 0);

roiManager("Rename", "ROITissus");

roiManager("Deselect");run("Select None");

setTool("point");

waitForUser("Wait on click", "Please click on the middle of a section and click on OK.");

getSelectionCoordinates(xclick, yclick);run("Select None");

print("Point du clic : "+xclick[0]);

print("click="+xclick[0]);

width=1024/nbBand;

print("Width="+width);

shift=(xclick[0]%width)-(width/2);

print("shift="+shift);

roiManager("Select", 0);

width=1024/nbBand;

if(shift>=0){

run("Specify...", "width="+width+" height=1024 x="+shift+" y=0 slice=1");

}

else{

run("Specify...", "width="+width+" height=1024 x="+(width+shift)+" y=0 slice=1");

print("x=x="+(width));

}

roiManager("Add");

if(shift!=0){nbBand=nbBand-1;}

for(i=1;i<=nbBand;i++){

if(shift>=0){

x=width*(i-1)+shift; }

else{

x=width*(i-1)+width+shift;}

print("xx="+x);

roiManager("Select", 1);

run("Specify...", "width="+width+" height=1024 x="+x+" y=0 slice=1");

roiManager("Update");

roiManager("Rename", "Largeband"+i);

roiManager("Select", newArray(0,1));

roiManager("AND");

run("To Bounding Box");

roiManager("Add");

roiManager("Deselect");

roiManager("Select", i+1);

roiManager("Rename", "Band"+i);

roiManager("Deselect");

}

roiManager("Select", 0);roiManager("Delete");

roiManager("Select", 0);roiManager("Delete");

roiManager("Show All with labels");

return nbBand;

}

function ApicalBasal(nomImage,i,pourcentApical,pourcentBasal){

setResult("Band", i, i+1);////

selectWindow(nomImage);

roiManager("Show None")

roiManager("Select", i);

getRawStatistics(nPixels, mean, min, max, std, histogram);

setResult("sum Band", i, nPixels*mean);////

setResult("mean Band", i, mean);////

Roi.getBounds(x, y, width, height);

makeRectangle(x, y, width, round(height*pourcentApical));

getRawStatistics(nPixels, mean, min, max, std, histogram);

setResult("mean Apical", i, mean);////

setResult("sum Apical", i, nPixels*mean);////

roiManager("Add"); roiManager("Select", roiManager("count")-1);

roiManager("Rename", "Band"+i+1+"_Apical");

roiManager("Select", i);

makeRectangle(x, y+round(height*(1-pourcentBasal)), width, round(height*pourcentBasal));

getRawStatistics(nPixels, mean, min, max, std, histogram);

setResult("mean Basal", i, mean);////

setResult("sum Basal", i, nPixels*mean);////

roiManager("Add");roiManager("Select", roiManager("count")-1);

roiManager("Rename", "Band"+i+1+"_Basal");

roiManager("Select", i);

makeRectangle(x, y+round(height*pourcentApical), width, round(height*(1-(pourcentApical+pourcentBasal))));

getRawStatistics(nPixels, mean, min, max, std, histogram);

setResult("mean Medial", i, mean);////

setResult("sum Medial", i, nPixels*mean);////

roiManager("Add");roiManager("Select", roiManager("count")-1);

roiManager("Rename", "Band"+i+1+"_Medial");

updateResults();

roiManager("Deselect");

}

function intensityMap(nomImage){

selectWindow(nomImage);

getDimensions(width, height, channels, slices, frames);

newImage("IntensityMap", "16-bit black", width, height, 1);

for(i=0;i<roiManager("count");i++){

selectWindow(nomImage);

roiManager("Select", i);

getStatistics(area, mean, min, max, std, histogram);

selectWindow("IntensityMap");

roiManager("Select", i);

setColor(mean);fill();

}

roiManager("Deselect");

run("Select None");

run("Fire");

run("Enhance Contrast", "saturated=0.35");

}
